# Supplementary material for: Tunable macroscale structural superlubricity in two-layer graphene via strain engineering
Source: Nat Commun. 2020 Mar 27;11:1595. doi: 10.1038/s41467-020-15446-y (PMC7101365; doi:10.1038/s41467-020-15446-y)
Supplement: Supplementary file 1 — Supplementary Information [file 41467_2020_15446_MOESM1_ESM.pdf]

Supporting Information

Tunable macroscale structural superlubricity in two-layer graphene via strain engineering

Androulidakis[1] et al.

[1]Institute of Chemical Engineering Sciences, Foundation of Research and Technology-Hellas (FORTH/ICE-HT), Stadiou Street, Platani, Patras, 26504 Greece
[2]Laboratory of Quantum and Computational Chemistry, Department of Chemistry, Aristotle University of Thessaloniki, GR-54124 Thessaloniki, Greece
[3]Department of Chemical Engineering, University of Patras, Patras 26504 Greece

[*]Corresponding author: c.galiotis@iceht.forth.gr or galiotis@chemeng.upatras.gr

Supplementary Note 1

AFM of the folded flake

In **Supplementary figure 1** an AFM image of the examined folded graphene by exfoliation is presented. During the mechanical testing, a discontinuity in the strain transfer was observed. The AFM image (taken before subjecting the sample to tension) revealed a pre-existing "wrinkle". The scan line crosses a wrinkle (denoted with the blue arrow in **Supplementary figure 1**) which acts like an edge, and the strain build-up starts from this point and towards the middle of the folded flake.

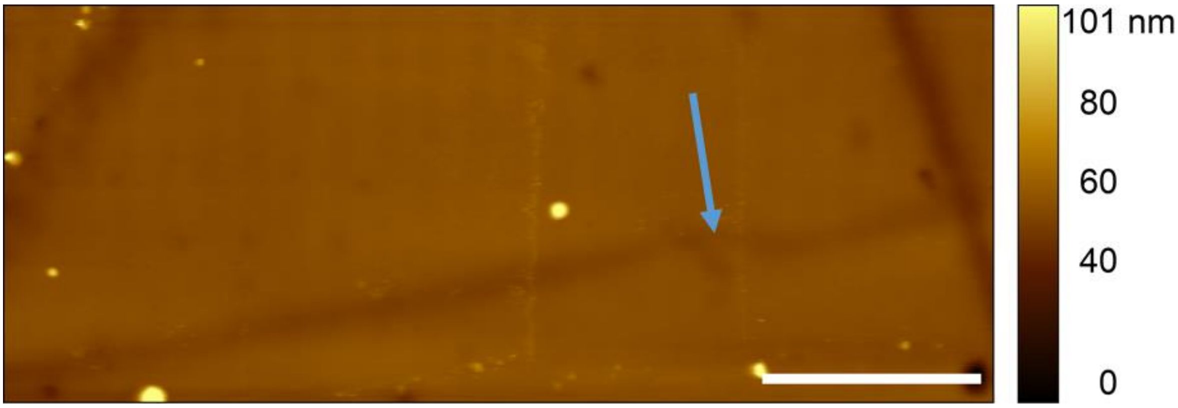

Supplementary Figure 1. Topography of the examined folded exfoliated bilayer. The presence of the wrinkle disrupts the strain transfer and acts like an edge. The scale bar is 5 microns.

Supplementary Note 2

CVD experiment

In **Supplementary figure 2** a schematic of the preparation procedure of the CVD-CVD bilayer graphene is presented. During CVD growth both sides of the copper foil are covered with graphene, and the graphene of one side is removed using oxygen plasma before transfer. Steps 2-4 describe the action taken for the deposition of the bottom graphene which is the crucial step. Steps 5-8 show the preparation of the second single

layer until its deposition on the top of the bottom larger single layer. The procedure is described in detail in the methods section in the main text.

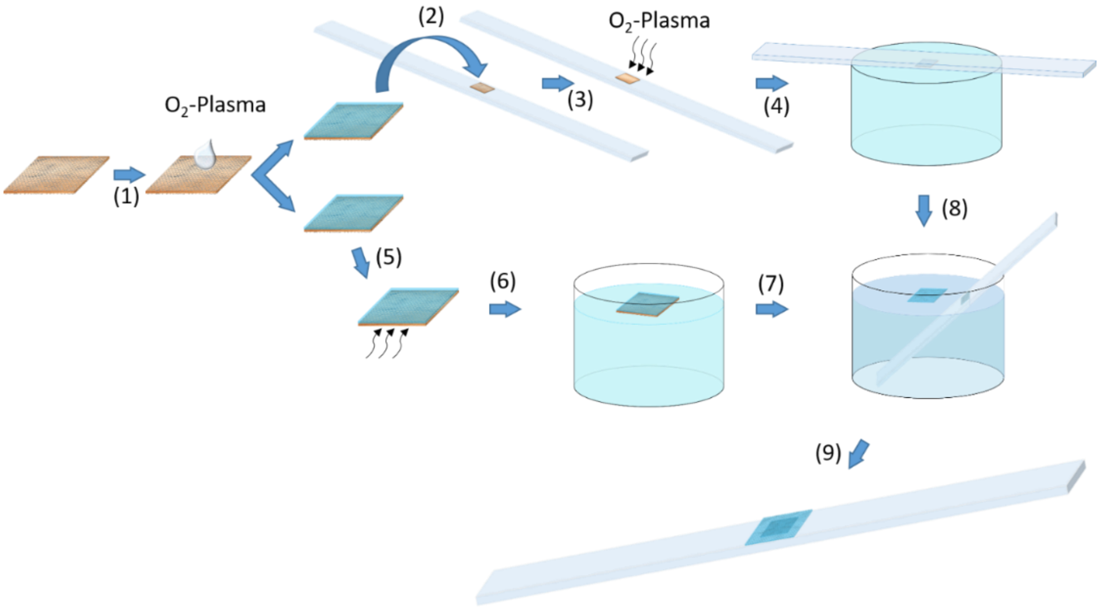

Supplementary Figure 2. Schematic of the steps followed for the fabrication of the CVD-CVD graphene sample. Detailed description of every step is given in the main text in the methods section.

We performed a second experiment on CVD/CVD bilayer in order to further confirm the results from the other two samples and more importantly to examine the flake at much larger area than the first. In **Supplementary figure 3** a schematic of the CVD-CVD sample is presented. The second graphene on the top was placed along with a thin layer of PMMA used for the transfer from the copper substrate. Because the substrate is from the same material, any attempt to remove this layer like an acetone bath, would affect/etch and the substrate. In order to avoid destroying the whole sample we did not remove this top thin PMMA layer.

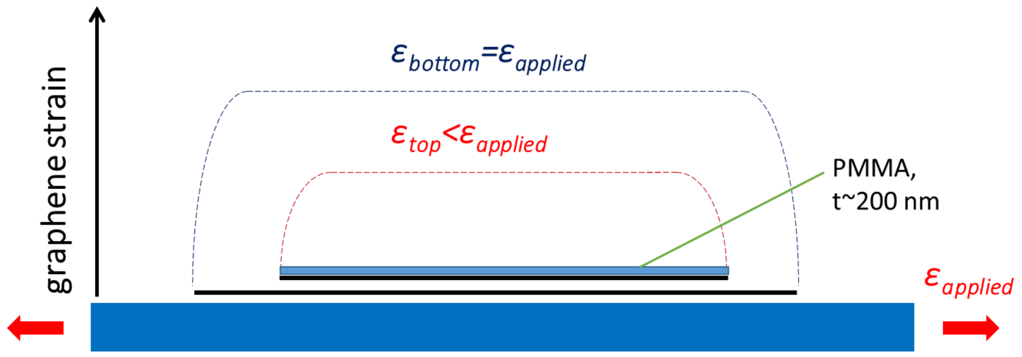

Supplementary Figure 3. Schematic of the sample of CVD-CVD graphene. A fragment of thin PMMA with thickness ~200 nm remained after the transfer.

In **Supplementary figure 4** the shift of the 2D peak in function with the applied strain presented for a second sample comprising of two CVD single layers. We note that this sample tested with a laser line of 785 nm, which is the reason for the different positions with the experiment presented in the main text. The shift rates are ~−16.9 cm^{-1}/% and ~−4.98 cm^{-1}/% for the bottom and top CVD layers, respectively. These shift rates are in very good agreement with the results in the main text, confirming the reproducibility of the experimental results.

Initially we scanned the sample close to the edge over a distance ~30 microns and at the strain level of ~0.50% a mapping line over 3 mm was performed in order to examine the behaviour of the CVD-CVD interface at the macroscale. The average values of the peak position from the small map (~30 microns) at 0.50 of tension are ~2592.9 cm^{-1} and 2611.49 cm^{-1} for the bottom and top CVD graphene, respectively. The corresponding averages obtained from the 3 mm scan presented in the main text are ~2593.95 cm^{-1} and 2611.63 cm^{-1}, respectively. The agreement is indeed excellent, confirming that the same behaviour holds for the whole sample.

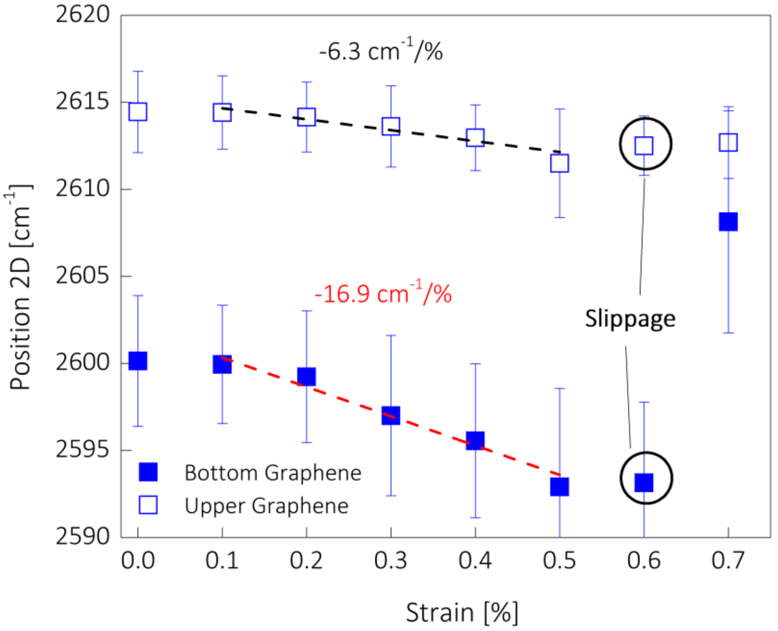

Supplementary Figure 4. The shift of the 2D peak of the bottom and top CVD single layer graphenes measured with a laser line of 785 nm.

Supplementary Note 3

MD simulations of wrinkled ILSS

To illustrate the effect of wrinkles on the interlayer shearing between two graphene layers, molecular dynamics simulations were performed, using the LCBOP potential[1] on supported bilayer graphene (as is the actual sample) that had been previously subjected to biaxial compressive strain of –0.6% in order to induce the formation of wrinkles. The compression was induced by reducing the computation cell (box) at a constant engineering strain rate of –0.005%/ps of its original dimensions, realized every 100fs. During the compressive stage the bottom layer was also at a temperature of 300K so thermal ripples were also imprinted in the overall corrugation, and kept rigid thereafter (for a comprehensive exposition on the validity of the rigid-substrate approach we refer to Ref. 2. The bilayer sheet is placed on an interacting plane (details are provided in the computational section of the main text). A large sheet was used in order to improve statistics of the forces acting on the atoms of the top layer. The dimensions of the top-layer

graphene are 40.0 nm x 10.0 nm, and thus has an area of 400 nm^2, and the bottom layer 40.0 nm x 39.8 nm. The top sheet was dragged at a constant velocity over the bottom layers that acted as a rigid corrugated substrate. The displacement of the top layer was performed with a small velocity of 0.02 Å/ps (which is in the range of the lowest used in the literature) and realized through the right-most edge (as shown in **Supplementary figure 6a**). Besides obtaining accurate averaged forces, by performing the simulation with a small velocity for the top-layer displacement, the top-layer followed the corrugations of the substrate without forceful detachments.

The interlayer shear stress is defined as the averaged dragging force over all atoms of the top-layer graphene divided by the nominal overlapping area of the top-layer graphene. In **figure Supplementary 6b** the evolution of the ILSS is shown with respect to the displacement of the top layer graphene with reference to it is right most edge (indicated at position *A* in **Supplementary figure 6a**). In **Supplementary figure 6c** a zoomed-in version of the plot shows repeating slip-and-stick patterns demonstrating that the fine structure of the interactions was also captured further affirming the quality of the results presented. We separate the simulation in distinct stages the start of which are depicted in **Supplementary figure 6a** and indicated by letters. Examination of the positions picked out in **Supplementary figure 6a** in conjunction with the plot of **Supplementary figure 6b** reveals significant ILSS drops as the top layer passes over wrinkled regions of the bottom layer. In the vicinity of position *A* the part of the bottom layer that is directly beneath the top layer is mostly flat. This results in the high ILSS values of ~55 MPa at displacements near position *A*, that are somewhat lower than the values (~68 MPa) of the respective fully flat case that we later examine. These values are in agreement with other computational results[2, 3]. The discrepancy with the experimental ILSS is due to the shearing direction and is discussed in the related section in the main text. At position *B* the top flake starts to pass over a fork present in the bottom layer that leads to a perpendicular (to the displacement

direction) wrinkle that starts forming at point *C*. Respectively, the ILSS starts to reduce and becomes significantly lower, with values ~25 MPa, once the top layer is fully above the perpendicular wrinkle. At position *D* the top layer has already mounted another fork of the bottom layer and has just started to also mount the second perpendicular wrinkle. This is imprinted in the ILSS pattern that continues to retain low values. At the final stage, noted at position *E*, the top layer gradually uncovers the second wrinkle (the *left* edge of the top layer is just starting to pass over the second wrinkle). As is by now expected, the ILSS starts to increase and eventually reaches the same high initial values. At any rate, we can safely conclude that the presence of wrinkles, either in the parallel or perpendicular direction, do not contribute to any increase of the ILSS. On the contrary, wrinkles or out-of-plane deformations due to the roughness of the substrate contribute to the lowering of the ILSS.

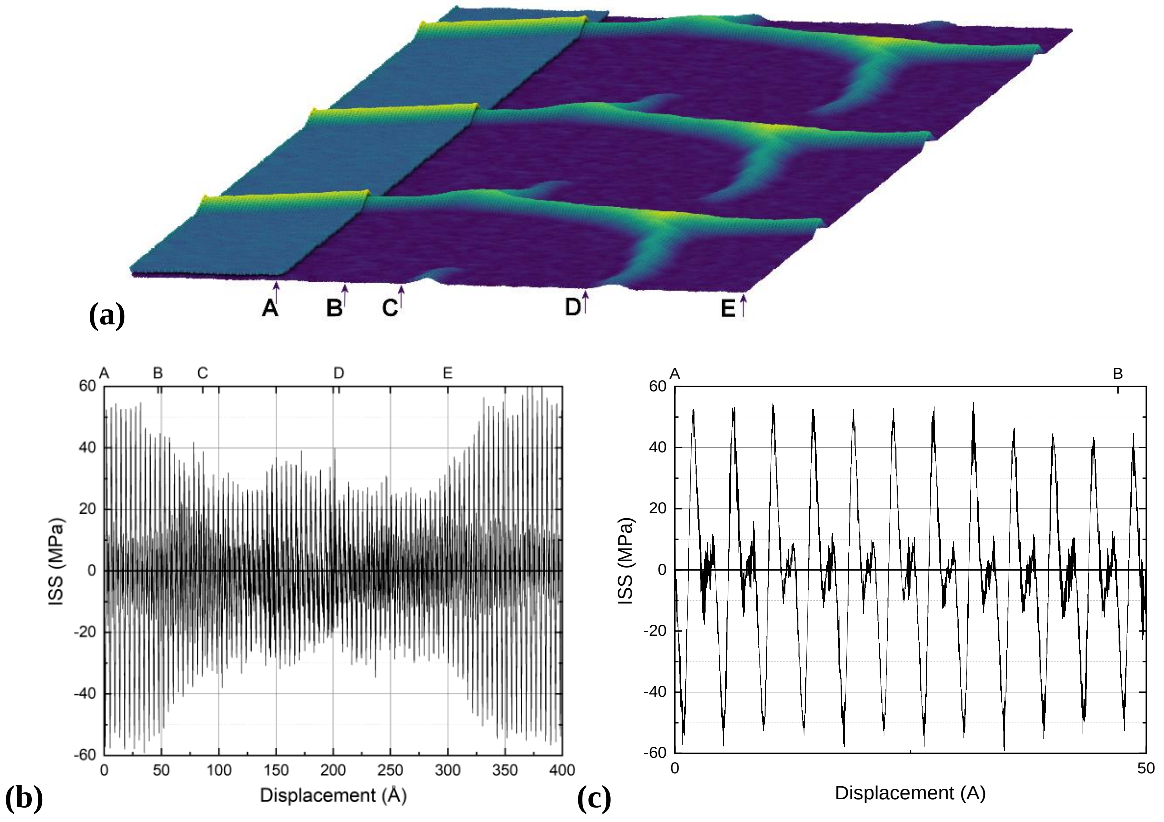

Supplementary Figure 5. (a) Wrinkled bilayer graphene. Three replications of the computational cell are shown at the initial state prior to shifting. The top layer is shifted along the *y*-axis (left to right as shown in figure). The dimensions of the bilayer sheet are 40.0 nm x 39.8 nm and has a total of 75978 atoms. The top layer has one fourth the overall

length along the *y*-axis. Simulations were performed using the LCBOP potential. (b) Evolution of the ILSS with respect to the displacement of the top layer graphene. (c) Zoomed-in version of the previous plot that clearly shows repeating slip & stick patterns.

The effect is also captured when the simulations are repeated employing the AIREBO potential[4], despite the significant differences in the formulation of the two potentials. The extent of the long-range Lennard–Jones interaction for the AIREBO potential were set to 3σ, which translates to 10.2Å. The AIREBO potential underestimates the lattice constant of graphene compared to LCBOP (and experiment), that leads to smaller areas of the graphenes by a factor of ~0.9688 at a temperature of 300K. Due to the much higher computational cost compared to the LCBOP potential, the simulation time was such to clearly demonstrate the ISS reduction when passing over the formed wrinkle perpendicular to the displacement direction. The graph of figure S6 reveals significant ILSS drops as the top layer passes over the wrinkled region of the bottom layer.

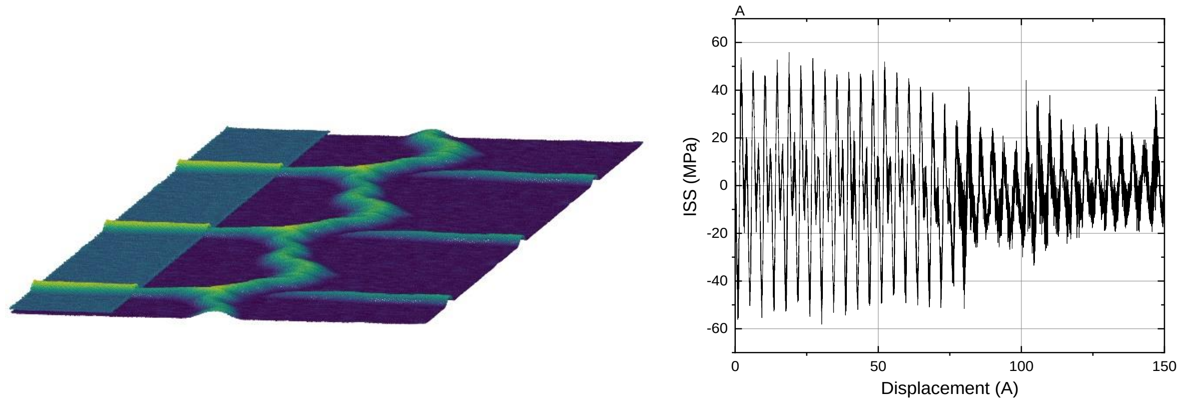

Supplementary Figure 6. (left) Wrinkled bilayer graphene. Three replications of the computational cell are shown at the initial state prior to shifting. The top layer is shifted along the y-axis (left to right as shown in figure). The dimensions of the bilayer sheet are 39.3 nm x 39.1 nm and has a total of 75978 atoms. The top layer has one fourth the overall length along the y-axis. Simulations were performed using the AIREBO potential. (right) Evolution of the ILSS with respect to the displacement of the top layer graphene.

As in the case of the LCBOP potential, the reduction in ILSS is also noted when using the AIREBO potential for chiral top layers. The results are shown in **Supplementary figure 7**.

The dimensions of the sheets that correspond to the LCBOP potential are provided in the **Supplementary table 1**. In the case of the AIREBO potential each dimension is scaled by a factor of 0.9843 due to the underestimation of the lattice constant.

Supplementary Table 1. Dimensions of the top layer used in each of the 'chiral' simulations when employing the LCBOP potential, number of atoms of the top layer, and overall number of atoms of the simulation cell.

Chiral Angle	L_x (nm)	L_y (nm)	Number of atoms Top Layer	Number of atoms Simulation Cell
**AC**	20.136	4.537	3608	19024
**ZZ**	7.160	4.543	1292	3960
**7.589°**	19.239	3.675	2736	9912
**15.295°**	14.488	4.182	2328	7756
**23.413°**	11.296	2.767	1204	5436

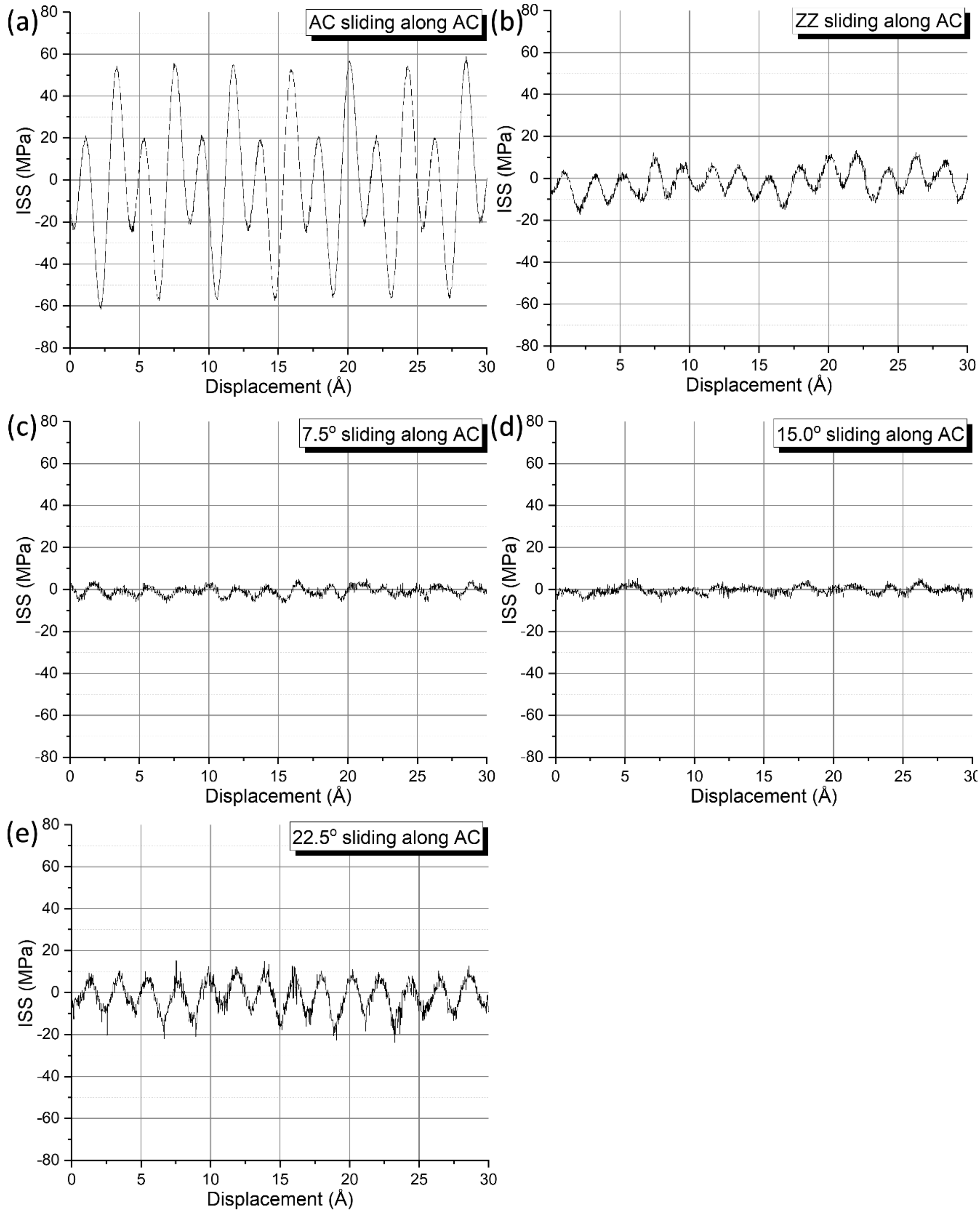

Supplementary Figure 7. ILSS values for various chiral directions at a temperature of 300 K. It is observed that when shearing a mono-layer graphene in achiral directions relative to the underline graphene, the stick-slip motion breaks down and the ILSS is significantly decreased. Performed employing the AIREBO potential.

3.3 Thermostat dampening.

The employed model and the usage of large cells produces clear results that are not masked by thermal fluctuations, as shown in **figure 6** of the main text and **Supplementary figures 5-7**. The temperature is controlled by means of a Nosé–Hoover thermostat with a temperature dampening parameter of T_{damp}=0.1ps. With this coupling strength the temperature is stabilized efficiently, as shown in **Supplementary figure 8**. To obtain some insight on possible dampening of the dynamics in connection with the temperature dampening strength[2,5-8], simulations were performed starting with a dampening parameter of T_{damp}=0.1ps which at some point during the simulation was switched to a larger value. Specifically, in **Supplementary figure 9a** the simulation starts with T_{damp}=0.1ps and after 500ps (one million time steps) switches to T_{damp}=1.0 ps, and the recorded ISS values remained unaffected. The same was observed for larger value of T_{damp}=4.0ps (**Supplementary figure 9b**). Usage of larger damping factors within this approach leads to inefficient energy dissipation.

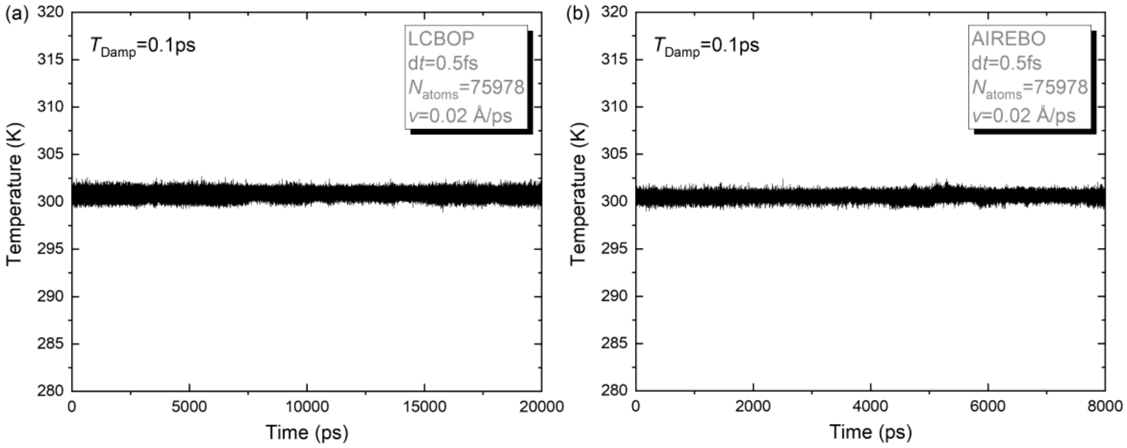

Supplementary Figure 8. Instantaneous temperatures the simulation of the top layer sliding over the bottom layer, using the (a) LCBOP and (b) AIREBO potential. The Nosé–Hoover thermostat dampening parameter was set to T_{damp}=0.1ps.

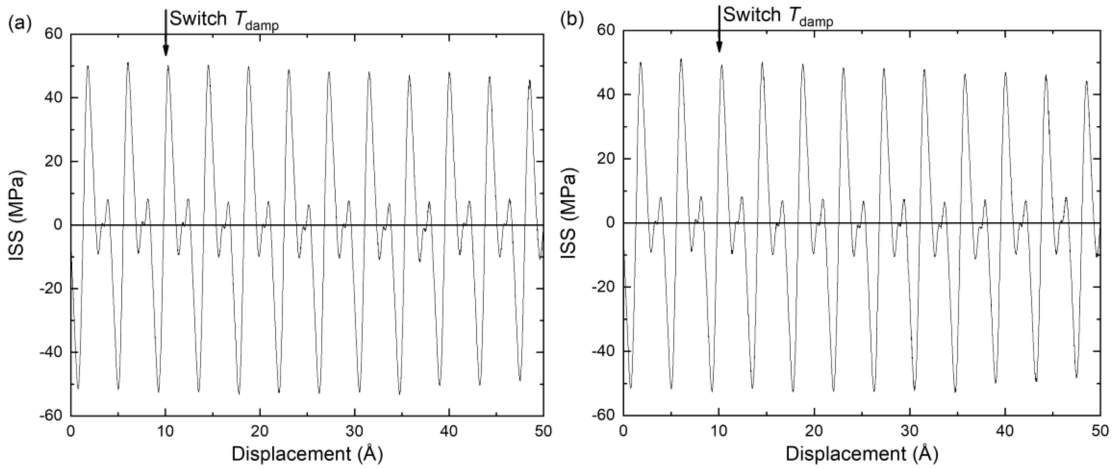

Supplementary Figure 9. Evolution of the ILSS with respect to the displacement of the top layer graphene. The Nosé–Hoover thermostat dampening parameter was switched from T_{damp}=0.1ps to (a) T_{damp}=1.0 ps, (b) T_{damp}=4.0 ps, after a displacement of 10 Å (i.e., after 500ps). Simulations were performed using the LCBOP potential.

Supplementary Note 4

Raman spectra of bilayer graphene folded and Bernal stacked

For comparison we present in the following figure the 2D Raman spectra of single layer, folded bilayer and Bernal stacked bilayer graphene. The full width half maximum (FWHM) of the Bernal stacked bilayer is ~53.8 cm^{-1}, while the corresponding values of the single layer and folded bilayer are ~30 cm^{-1} and ~20 cm^{-1}, respectively. The Bernal stacked bilayer is fitted with very good accuracy with four Lorentzian curves as seen in **Supplementary figure 10a**. If we fit the spectrum of the folded bilayer the curves tend to overlap, thus not providing any additional information. As discussed in the main text, fitting with two Lorentzian curves provides very good fitting and giving accurate estimated of the strain, which is also validated by the shift rate of the 2D peak.

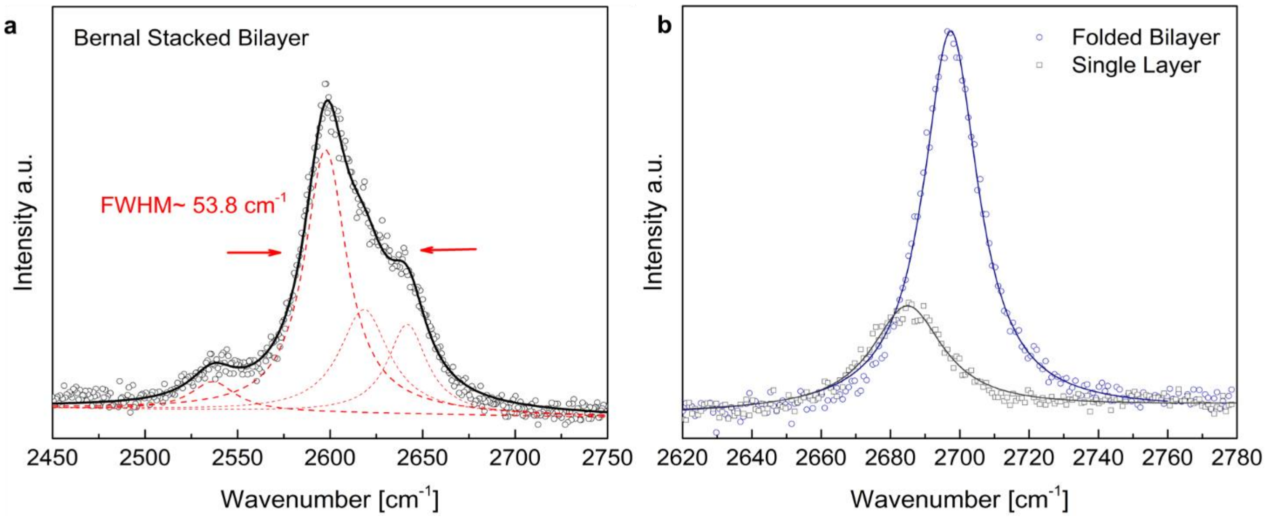

Supplementary Figure 10. (a) The 2D peak of a Bernal stacked bilayer fitted with four Lorentzian curves. (b) The 2D peak of a single layer and a folded bilayer as presented in the main text.

References

1. Los, J.; Fasolino, A., Intrinsic long-range bond-order potential for carbon: Performance in Monte Carlo simulations of graphitization. *Physical Review B* **2003,** *68* (2), 024107.
2. Song, Y.; Mandelli, D.; Hod, O.; Urbakh, M.; Ma, M.; Zheng, Q., Robust microscale superlubricity in graphite/hexagonal boron nitride layered heterojunctions. *Nature materials* **2018,** *17* (10), 894.
3. Wei, X.; Naraghi, M.; Espinosa, H. D., Optimal length scales emerging from shear load transfer in natural materials: application to carbon-based nanocomposite design. *Acs Nano* **2012,** *6* (3), 2333-2344.
4. Stuart, S. J.; Tutein, A. B.; Harrison, J. A., A reactive potential for hydrocarbons with intermolecular interactions. *The Journal of chemical physics* **2000,** *112* (14), 6472-6486.
5. Vanossi, A.; Manini, N.; Urbakh, M.; Zapperi, S.; Tosatti, E., Colloquium: Modeling friction: From nanoscale to mesoscale. *Reviews of Modern Physics* **2013,** *85* (2), 529-552.
6. Basconi, J. E.; Shirts, M. R., Effects of Temperature Control Algorithms on Transport Properties and Kinetics in Molecular Dynamics Simulations. *Journal of Chemical Theory and Computation* **2013,** *9* (7), 2887-2899.
7. Smith, E. D.; Robbins, M. O.; Cieplak, M., Friction on adsorbed monolayers. *Physical Review B* **1996,** *54* (11), 8252-8260.
8. Tomassone, M. S.; Sokoloff, J. B.; Widom, A.; Krim, J., Dominance of Phonon Friction for a Xenon Film on a Silver (111) Surface. *Physical Review Letters* **1997,** *79* (24), 4798-4801.
